# PANACEA: network-based methods for pharmacotherapy prioritization in personalized oncology

**DOI:** 10.1093/bioinformatics/btad022

**Published:** 2023-01-12

**Authors:** Ege Ulgen, Ozan Ozisik, Osman Ugur Sezerman

**Affiliations:** Department of Biostatistics and Medical Informatics, School of Medicine, Acibadem Mehmet Ali Aydinlar University, Istanbul 34752, Turkey; Aix Marseille University, Inserm, MMG, Marseille 13385, France; Department of Biostatistics and Medical Informatics, School of Medicine, Acibadem Mehmet Ali Aydinlar University, Istanbul 34752, Turkey

## Abstract

**Motivation:**

Identifying appropriate pharmacotherapy options from genomics results is a significant challenge in personalized oncology. However, computational methods for prioritizing drugs are underdeveloped. With the hypothesis that network-based approaches can improve the performance by extending the use of potential drug targets beyond direct interactions, we devised two network-based methods for personalized pharmacotherapy prioritization in cancer.

**Results:**

We developed novel personalized drug prioritization approaches, PANACEA: PersonAlized Network-based Anti-Cancer therapy EvaluAtion. In PANACEA, initially, the protein interaction network is extended with drugs, and a driverness score is assigned to each altered gene. For scoring drugs, either (i) the ‘distance-based’ method, incorporating the shortest distance between drugs and altered genes, and driverness scores, or (ii) the ‘propagation’ method involving the propagation of driverness scores via a random walk with restart framework is performed. We evaluated PANACEA using multiple datasets, and demonstrated that (i) the top-ranking drugs are relevant for cancer pharmacotherapy using TCGA data; (ii) drugs that cancer cell lines are sensitive to are identified using GDSC data; and (iii) PANACEA can perform adequately in the clinical setting using cases with known drug responses. We also illustrate that the proposed methods outperform iCAGES and PanDrugs, two previous personalized drug prioritization approaches.

**Availability and implementation:**

The corresponding R package is available on GitHub. (https://github.com/egeulgen/PANACEA.git).

**Supplementary information:**

Supplementary data are available at *Bioinformatics* online.

## 1 Introduction

Cancer is a heterogeneous collection of many distinct diseases that possess numerous capabilities, so-called ‘Hallmarks of Cancer’ acquired during their development (Hanahan, 2022; Hanahan and Weinberg, 2000, 2011). Somatic mutations that alter the function of crucial cancer genes, called driver genes, acquired during an individual’s lifetime are the basis of cancer formation and progression (Stratton *et al.*, 2009). As such, the advancement of sequencing technologies during the past decade transformed the field of oncology by identifying essential alterations, genes and tumorigenic processes (Garraway and Lander, 2013; Kamalakaran *et al.*, 2013; Stratton, 2011). Importantly, these technologies allowed individualized analysis of cancer genomes, resulting in more precise diagnosis and tailored treatments.

Identifying the most appropriate pharmacotherapy options based on genomic events is still a significant challenge in personalized oncology. However, computational methods for prioritizing drugs for individual patients using solely genomics data are underdeveloped.

In a landmark study in 2015, Rubio-Perez *et al.* demonstrated that while a minority of cancer patients were treatable by following the clinical guidelines at the time, many more could benefit from the *in-silico* drug prescription strategy they proposed (Rubio-Perez *et al.*, 2015). This *in-silico* drug prescription strategy was based on identifying drivers in each tumor and their druggability options, considering direct targeting, indirect targeting and gene therapies. This study demonstrated the potential benefit of utilizing genomics information for individualized drug prioritization in oncology.

Following the study by Rubio-Perez *et al.*, other studies developed methods for personalized drug therapy identification using genomics data. Two such prominent approaches are iCAGES and PanDrugs. iCAGES is a framework that analyzes personal cancer genomes to prioritize variants, genes and drugs (Dong *et al.*, 2016). The drug prioritization approach (Layer 3) of iCAGES calculates the joint probability for each drug being the most effective (iCAGES drug score) by integrating three scores for a given drug: (i) iCAGES gene scores (reflecting the probability of being a driver) for the drug’s direct/indirect target, (ii) normalized BioSystems (Geer *et al.*, 2010) probability measuring the maximum relatedness of the drug’s direct target with each mutated gene (final target) and (iii) the drug’s marginal activity score from PubChem (Wang *et al.*, 2017), measuring the bioactivity of the drug. PanDrugs is a method to identify potentially druggable molecular alterations and prioritize drugs in an individual tumor sample using genomics findings (Piñeiro-Yáñez *et al.*, 2018). PanDrugs categorizes druggable genes as (i) direct targets, (ii) biomarkers and (iii) pathway members. To prioritize drug therapy for individual tumors, PanDrugs utilizes a gene score that assesses the input genes’ biological significance in cancer and its clinical importance (GScore), as well as a drug score that estimates drug response and treatment suitability (DScore). GScore considers gene essentiality and tumoral vulnerability, gene relevance in cancer, the biological impact of mutations, the frequency of gene alterations and their clinical implications; DScore considers drug indication and status, gene–drug associations and number of hits. PanDrugs recommends the ‘Best Candidate Therapy’ list based on the accumulated and weighted scoring of GScore and DScore. It should be noted that neither iCAGES nor PanDrugs utilize interaction information, which we propose can further improve accurate prioritization of drugs, beyond direct drug–target gene interactions and, in the case of PanDrugs, pathway membership information.

There is growing interest in network-based methods for drug repurposing/repositioning in cancer (Turanli *et al.*, 2021). Network-based methods have also been successfully used to infer personalized drug targets (Shnaps *et al.*, 2016) and prioritize candidate cancer drugs in the context of drug repurposing against specific types of cancer (Di *et al.*, 2019). While some such approaches could be extended for personalized drug prioritization, prior work on network-based methods for personalized drug prioritization is limited. We propose that network-based approaches have the potential to improve the performance of personalized drug prioritization by extending the use of potential drug targets beyond direct interactions that prior methods utilize.

This study aimed to develop novel personalized drug prioritization approaches utilizing network-based methods, collectively named PANACEA: PersonAlized Network-based Anti-Cancer therapy EvaluAtion. Using somatic genomic alterations, we devised two methods that are run on a protein interaction network (PIN) extended with drugs: (i) the ‘distance-based’ method, incorporating the shortest distance between drugs and altered genes as well as the driverness scores derived from driveR (Ülgen and Sezerman, 2021), (ii) the ‘propagation’ method involving network propagation of driverness scores to the drug nodes via a random walk with restart (RWR) framework. In order to demonstrate PANACEA methods and compare them against two previous methods, namely iCAGES and PanDrugs, we used data from three sources. First, we applied the PANACEA methods using The Cancer Genome Atlas (TCGA) data and demonstrated that the top-ranking drugs are relevant for pharmacotherapy in cancer. Secondly, using Genomics of Drug Sensitivity in Cancer (GDSC) data, we evaluated the top-ranking drugs in a large number of cancer cell lines by assessing drug–response data, illustrating that the majority of cell lines are indeed sensitive to the prioritized drug treatments. Lastly, we applied the PANACEA methods on two cases with known drug responses and demonstrated that PANACEA could recommend the effective drugs. In the comparisons, the PANACEA methods performed as well as or better than the prior personalized approaches that utilize somatic genomics data, iCAGES and PanDrugs.

## 2 Materials and methods

### 2.1 Overview

**Fig. 1.**
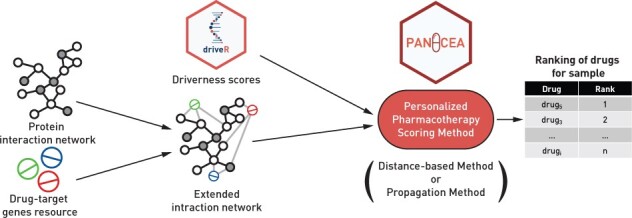
Overview of the personalized pharmacotherapy scoring methods

This study aimed to devise network-based methods for scoring pharmacotherapy options for a single tumor sample using genomics information. Each approach, described in detail in the following section, begins by assigning the ‘driverness’ scores (reflecting the probability that the given gene is a driver gene) of all altered [i.e. underwent either with somatic mutation or somatic copy-number alteration (SCNA)] genes in the given sample onto an undirected PIN, which is extended by drug–target interactions (Fig. 1). Next, either the ‘distance-based’ or the ‘propagation’ method is used to score and rank the drugs from the drug–target genes resource for the given sample. These network-based methods are described in detail in the relevant sections. Briefly, the ‘distance-based’ method assigns a score to each drug based on the driver genes’ scores and the length of the shortest path between the drug and each driver gene, whereas the ‘propagation’ method scores drugs by propagating driver scores through the network via an RWR framework.

The PANACEA methods were evaluated using data from (i) TCGA, (ii) GDSC (Yang *et al.*, 2013) and (iii) two cancer cases with known drug response. The evaluation approaches are described in detail in the following sections.

### 2.2 Determination of ‘Driverness’ scores

For determining the driverness scores of all altered genes in each sample, our previously proposed method for prioritizing cancer driver genes, driveR (v0.3.0), was used (Ülgen and Sezerman, 2021). This method utilizes somatic genomics data to prioritize driver genes in personalized/batch analyses. Briefly, driveR processes somatic mutations and somatic copy-number alterations SCNAs to establish 26 features for each altered gene, including the maximum coding impact, the maximum non-coding impact, a proxy SCNA score, whether the gene contains a hotspot mutation or a double-hit (copy-number loss + mutation), literature-association score via Phenolyzer (Yang *et al.*, 2015) for the given cancer type and presence in 21 cancer-related KEGG pathways (Kanehisa *et al.*, 2021). These features are then used to obtain ‘driverness’ score via the cancer-type specific sub-model of the pre-trained multi-task learning classification model.

### 2.3 PIN and extension by drugs

All methods were performed using the STRING v11.5 PIN (Szklarczyk *et al.*, 2019). For moderate confidence, interactions with a combined score of <0.4 were discarded.

To demonstrate that the methods are robust to the change of PIN, all analyses on TCGA data were also performed and evaluated using the BioGRID 4.4.205 PIN (Oughtred *et al.*, 2021); these results are provided as Supplementary Material.

PANACEA incorporates drug–target gene information by extending the PIN with drugs: the drugs are added as nodes connected to their target genes. This network will be referred to as the ‘extended PIN’. For this study, directionality of interactions was not considered.

In our analyses on GDSC data, we extended the PIN using the drugs available in the GDSC data. In our remaining analyses, we extended the PIN using drug–gene interactions from expert-curated sources on DGIdb (May 2021) (Freshour *et al.*, 2021), containing direct interactions: CancerCommons, CGI, ChemblInteractions, CIViC, ClearityFoundationBiomarkers, ClearityFoundationClinicalTrial, COSMIC, DoCM, MyCancerGenome, MyCancerGenomeClinicalTrial, TALC, TdgClinicalTrial and TEND. In all analyses, drugs targeting completely overlapping sets of genes were merged and considered as a single drug during evaluation.

### 2.4 PIN and extension by drugs

In PANACEA, we propose two different methods for personalized pharmacotherapy scoring, described below.

#### 2.4.1 Distance-based method

For the distance-based method, initially, the ‘driverness’ scores of all genes with somatic genomic alterations are mapped onto the extended PIN. A score incorporating ‘driverness’ score and distance between the drug and each gene is calculated for each drug–gene pair, as formulated in Equation (1).

(1) $$s\left(d,g\right)=\frac{1}{{\left(\mathrm{dist}\left(d,g\right)+1\right)}^{2}}{\mathrm{driver}}_{g},$$

where *d* is the drug, *g* is an altered gene, dist(*d, g*) is the distance of the shortest path between *d* and *g* and driver_*g*_ is the driveR score for *g*. In Equation (1), the denominator includes the addition of one to prevent division by zero, and is raised to the power of two for a faster decay of the effect as the distance increases, putting more emphasis on genes closer to the drug. We note that drug nodes are never used as intermediary nodes in the calculation of shortest paths, as the interactions between drugs and their targets are considered unidirectional.

The aggregated score for the drug is then calculated using Equation (2).

(2) $$\mathrm{score}\left(d\right)=\frac{1}{\left|\mathrm{genes}\right|}\sum_{g\in \mathrm{genes}}^{}s\left(d,g\right),$$

where genes is the set of altered genes. Only altered genes with a ‘driverness’ score >0.05 were used to consider more impactful genes and to eliminate the detrimental effect of potentially irrelevant genes that do not contribute to ‘driverness’, also shortening the run-time of the calculations.

#### 2.4.2 Propagation method

Initially, the driverness scores of all genes with somatic genomic alterations are mapped onto the extended PIN. Using the driverness scores as prior knowledge (*Y*), the RWR framework propagates this information. First, the adjacency matrix (*W*) of the extended PIN is normalized using Laplacian normalization as $W^{\prime }=D^{-1/2}WD^{-1/2}$, where *D* is a diagonal matrix such that $D_{ii}=\sum_{j}W_{ij}$. To compute the propagation scores *F*, the following iterative procedure is used: starting with $F^{\left(0\right)}=Y$, *F* is updated at iteration *t* as follows:

(3) $$F^{\left(t\right)}={\left(1-\alpha \right)\;W'F}^{\left(t-1\right)}+\alpha \;Y,$$

where *α* is the restart parameter. The iterative procedure is repeated until convergence when:

(4) $${\left\|F^{\left(t\right)}-F^{\left(t-1\right)}\right\|}_{2}<10^{-4},$$

or until the number of iterations exceeds 1000 (median number of iterations =10 for TCGA THCA-US data, *n* = 486, on STRING PIN, range =4–21). The procedure for the selection of *α* is described in the following section. The score of a drug is its final propagation score.

### 2.5 Analysis of TCGA data

#### 2.5.1 Selection of cohorts

To determine the propagation method’s restart parameter and evaluate the methods, we aimed to select two dissimilar datasets from TCGA. For this purpose, first, somatic mutation and SCNA data for all cohorts available on the International Cancer Genome Consortium data portal were obtained (Zhang *et al.*, 2011). The ‘driverness’ scores of altered genes for each sample in each cohort were determined using driveR (v0.3.0). Next, the median driveR score of each gene in each cohort was determined. Finally, to assess the similarity between cohorts, Spearman’s rank correlation coefficients (of median scores of common genes) between each pair of cohorts were calculated to cluster the cohorts (Supplementary Fig. S1). The minimum correlation was observed between LAML-US (acute myeloid leukemia, *n* = 135) and THCA-US (thyroid cancer, *n* = 486). Hence, LAML-US was used for parameter selection, while THCA-US was used to evaluate the methods’ performances.

#### 2.5.2 Selection of the restart parameter (*α*)

For the propagation method, to determine the restart parameter, *α*, the LAML-US cohort was used to rank drugs per each sample using *α* = 0.05, 0.1, 0.25, 0.5, 0.75 and 0.9 and the distributions of the supported drug proportions (Tiers 1–5) in Top 5, 10 and 25 were assessed (Supplementary Fig. S2 and Supplementary Table S1). Following manual inspection of relevance of the results, the optimal *α* was determined to be 0.05. This value of *α* was used for all analyses using the propagation method.

#### 2.5.3 Evaluation

For evaluating the relevance of each method’s ranking, we first classified each drug into one of the following tiers:

- Tier 1A: approved for the cancer type under study
- Tier 1B: approved for other cancer types
- Tier 2A: in clinical trial(s) for the cancer type under study
- Tier 2B: in clinical trial(s) for other cancer types
- Tier 3A: curated for the cancer type under study in Comparative Toxicogenomics Database (CTD) (Davis *et al.*, 2021)
- Tier 3B: curated for other cancer types in CTD
- Tier 4A: inferred for the cancer type under study in CTD
- Tier 4B: inferred for other cancer types in CTD
- Tier 5: repurposed for cancer [in ReDO_DB (Pantziarka *et al.*, 2018)]
- Tier 6: no support.

Approved drugs and drugs in clinical trials were obtained using the R package oncoPharmaDB (v0.6.6), which uses data from the Open Targets Platform (Ochoa *et al.*, 2021). For Tiers 3 and 4, data for MeSH terms d002277 (carcinoma) and d009369 (neoplasm) were retrieved on December 16, 2021, from the CTD (Davis *et al.*, 2021). For drugs repurposed for cancer, ReDO_DB (November 24, 2021 update) was used (Pantziarka *et al.*, 2018).

For these sources, drug name standardization for compatibility with DGIdb drugs was performed via the PubChem Identifier Exchange Service (https://pubchem.ncbi.nlm.nih.gov/idexchange/idexchange.cgi).

To assess the relevance of the selected drugs, for each tumor sample, the Top 5, 10 and 25 drugs as ranked by each method were identified. We should note that the set of top *n* drugs additionally contains the drugs that have tying scores with the top *n*. For example, if the sixth and seventh drugs’ scores were the same as the fifth one, the sixth and the seventh drugs were also included in the list of Top 5. After the tier of each drug [i.e. whether the drug is FDA-approved for the current cancer type (Tier 1A), whether the drug is FDA-approved for any other cancer type (Tier 1B) or whether it is in clinical trial(s) (Tier 2) etc., see above] was determined, the proportions of drugs in each tier were calculated for top *n* drugs. Finally, these proportions per sample were assessed to observe the level of support for the drugs selected for each patient (i.e. comparing the cumulative proportion of Tiers 1–5 versus Tier 6).

Moreover, the THCA-US samples were also analyzed with iCAGES and PanDrugs. For iCAGES, the Top 10 drugs were selected for each sample whereas the ‘Best Candidate Therapy’ was used for PanDrugs. The drugs selected by each approach (i.e. PANACEA distance-based top 10, PANACEA propagation top 10, iCAGES top 10 and PanDrugs ‘Best Candidate Therapy’) were then compared in terms of relevance for oncotherapy via paired Wilcoxon tests.

Additionally, the proportions of samples with any supported drugs that directly target the driver gene(s) for each sample were calculated. This proportion was then compared with the proportion of supported (Tiers 1–5) drugs selected by each novel method.

To assess the discriminative capacity of the PANACEA methods, the proportion of drugs indicated for the given cancer type (Tiers 1A, 2A, 3A and 4A) within the Top 10 selection was calculated for each sample.

### 2.6 Analysis of GDSC data

#### 2.6.1 Data

For the GDSC analyses, dose–response data for Sanger GDSC1 and GDSC2 (February 2020) were obtained from the GDSC website (https://www.cancerrxgene.org/) (Iorio *et al.*, 2016; Picco *et al.*, 2019). Cancer Cell Line Encyclopedia mutation, copy-number alteration and sample information data were obtained from DepMap Public 21Q4 release (Barretina *et al.*, 2012). DGIdb was used as the drug–target genes resource. For GDSC drugs that were not found in DGIdb, target genes were obtained from the GDSC website. Only cell lines of 18 cancer types compatible with analysis via driveR were included in the analyses, resulting in 515 cancer cell lines.

#### 2.6.2 Evaluation

Both PANACEA methods were performed and the Top 10 drugs (including ties) per each method for each cell line were determined. For each drug, the area under the drug–response curve (AUC), reflecting drug–response level, was available. In the drug–response curve, the administered dose is displayed on the *X*-axis versus the response on the *Y*-axis. Thus, higher AUC indicates better response. The AUC values of these selected drugs (for all common cell lines for which at least one drug is recommended) were compared against two naïve methods via paired Wilcoxon tests: (i) selecting drugs that directly target driver genes for the given sample and (ii) selecting drugs that directly target driver genes or direct neighbors of these driver genes for the given sample.

Additionally, iCAGES and PanDrugs analyses were performed on all available cell line data to rank the drugs for each cell line sample. The top drug (if any recommended by all methods) selected by each method (PANACEA distance-based, PANACEA propagation, iCAGES and PanDrugs) was then compared via paired Wilcoxon tests.

### 2.7 Cases with known drug response

iCAGES and PanDrugs are widely used personalized drug prioritization methods that use somatic genomics data as input, similar to PANACEA. For further comparing the performance of the PANACEA methods with these approaches, two cases with known drug response were analyzed: (i) a bladder cancer (BLCA) with an extraordinary response to everolimus and pazopanib (Wagle *et al.*, 2014), and (ii) a sorafenib-sensitive lung adenocarcinoma (LUAD) case (Imielinski *et al.*, 2014).

Somatic whole exome sequencing results, including somatic mutation and SCNA, were obtained from the corresponding articles for both cases. Using driveR results, the PANACEA methods were utilized to rank DGIdb drugs, and whether or not the effective drugs were selected by each method within Top 10 were determined. Additionally, driveR results were saved as gene ranking files to be used as input for PanDrugs, and pan-cancer analyses were performed using PanDrugs. The iCAGES drug prioritization results for both cases were obtained from the additional files of the original article (namely, Additional files 12 and 13). Whether or not the effective drugs were recommended (i.e. whether the effective drugs were within Top 10 for PANACEA methods, PanDrugs DScore and iCAGES, and ‘Best Candidate Therapy’ list by PanDrugs) was assessed across all methods.

## 3 Results

### 3.1 TCGA analysis

The distance-based method and the propagation method were initially established and evaluated using the two selected TCGA cohorts: LAML-US (acute myeloid leukemia, *n* = 135) was used for parameter selection, and the methods were assessed using THCA-US (thyroid cancer, *n* = 486).

For THCA-US, the distributions of the number of drugs for each sample in the Top 5, 10 and 25 (including ties) for each method are presented in Supplementary Figure S3. Of note, the distance-based method had a large variability in terms of the number of selected drugs (due to a large number of drugs with tying scores).

**Fig. 2.**
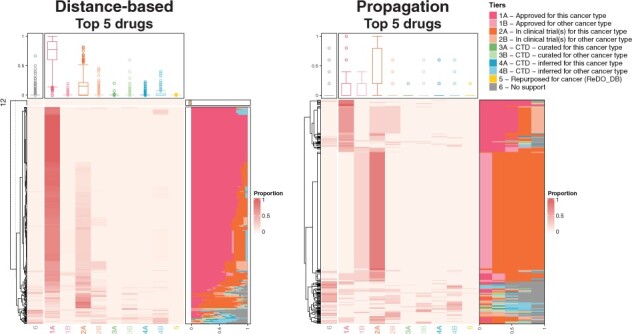
Heatmaps of proportions of selected (Top 5) drugs in each tier for each method (THCA-US data, STRING PIN). For each heatmap, rows are for samples, and columns are for drug tiers. Right-hand row-side stacked bar plot displays the proportion of selected drugs in each tier per sample. Top box plots show the distributions of proportions of selected drugs per tier. The legend for drug tiers is provided on the top right. There were no genes with driveR score >0.05 for 12 samples, separately displayed in the dendrogram for the distance-based method

Within each selection (i.e. Top 5, 10 or 25), the relevance of selected drugs to cancer therapy was evaluated via annotation of drug tiers, as outlined in Section 2. Heatmaps displaying the proportions of the Top 5 drugs in each tier per sample are presented in Figure 2. Heatmaps of the proportions of the Top 10 and 25 drugs in each tier per sample are provided in Supplementary Figure S4. For both the distance-based and propagation methods, almost every sample had at least one supported drug (Tiers 1–5) in the Top 5. For the distance-based method, the proportion of supported drugs within the Top 5 was over 0.75 for 96.2% of samples. Additionally, the proportion of supported drugs within the Top 5 was over 0.9 for 90.5% of all samples. Similarly, for the propagation method, 91.15% of samples had a proportion of supported drugs over 0.75, whereas 78.4% had a proportion over 0.9 supported drugs in the Top 5.

Using the two naïve approaches, by (i) selecting all drugs that directly target a sample’s driver gene(s) and (ii) selecting all drugs that target a sample’s driver gene(s) or its direct neighbors, at least one Tier 1–5 drug was recommended to 75.51% of all samples, and at least one Tier 1–2 drug was recommended to 74.69% (Table 1). The percentages of samples that were recommended at least one Tier 1–5 drug, as well as Tier 1–2 drug, were substantially higher for the proposed PANACEA methods.

**Table 1.** Percentages of samples with Tier 1–5 or Tier 1–2 drugs prioritized by naïve and PANACEA methods

	Tiers 1–5 (%)	Tiers 1–2 (%)
Directly targeting	75.51	74.69
Directly or adjacent targeting	75.51	74.69
PANACEA distance-based method	97.53	96.91
PANACEA propagation method	100.00	96.50

*Note*: ‘Directly targeting’ indicates the naïve method in which all drugs directly targeting the sample’s driver gene(s) were selected. ‘Directly or Adjacent Targeting’ indicates the naïve method where all drugs directly targeting the sample’s driver gene(s) or their direct neighbors were selected. For the PANACEA methods, the Top 5 drugs were selected.

**Fig. 3.**
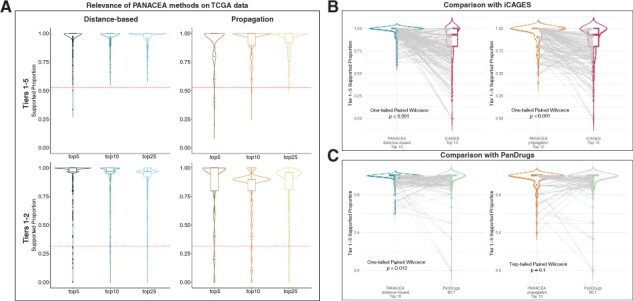
Assessment of relevance of selected drugs. (**A**) Violin and boxplots, displaying the distribution of proportions of supported (either Tiers 1–5 or Tiers 1–2) selected drugs per drug prioritization method (THCA-US data). Dashed red lines indicate the overall/expected proportion of Tier 1–5 and Tier 1–2 drugs in DGIdb. (**B**) Comparison of selected drugs between PANACEA methods—Top 10 and iCAGES—Top 10. (**C**) Comparison of selected drugs between PANACEA methods Top 10 and PanDrugs—best candidate therapy

Figure 3A displays the distributions of the proportion of selected drugs in Tiers 1–5 (all supported tiers) and Tiers 1–2 (approved/clinical trial drugs) for each method. For both methods, the median proportions of Tier 1–5 drugs were significantly higher than the overall proportion of Tier 1–5 drugs in DGIdb (overall proportion =0.53, all Wilcoxon tests *P* < 0.001) for all Top 5, 10 and 25. Similarly, the median proportions of Tier 1–2 drugs were higher than the overall proportion of Tier 1–2 drugs in DGIdb (overall proportion =0.31, all Wilcoxon tests *P* < 0.001). When the two PANACEA methods were compared, the Top 10 selection of the ‘distance-based’ method was observed to yield a significantly higher proportion of supported drugs (Tiers 1–5) compared to the Top 10 selection of the ‘propagation’ method (one-tailed paired Wilcoxon test *P* < 0.001).

In the comparisons of PANACEA methods with iCAGES and PanDrugs, PANACEA methods outperformed iCAGES using both distance-based and propagation methods, and they outperformed PanDrugs using the distance-based method. When the relevance of drugs selected by PANACEA methods (Top 10) was compared with those selected by iCAGES (Top 10), it was observed that the proportion of supported (oncotherapy-associated Tiers 1–5) drugs was significantly higher for PANACEA methods compared to iCAGES (one-tailed paired Wilcoxon tests *P* < 0.001, Fig. 3B). Similarly, the Top 10 drugs by the PANACEA distance-based method were significantly more relevant to oncotherapy compared to the ‘best candidate therapy’ drugs selected by PanDrugs (one-tailed paired Wilcoxon tests *P* = 0.012, Fig. 3C, left). The supported proportion of drugs within the Top 10 drugs by the PANACEA propagation method was not significantly different from the supported proportion of the ‘best candidate therapy’ drugs selected by PanDrugs (two-tailed paired Wilcoxon tests *P* = 0.1, Fig. 3C, right).

For both methods, the distributions of frequencies of drugs observed in the Top 5 in each THCA-US sample were right-skewed (Supplementary Tables S2 and S3), implying that most drugs in the Top 5 are specific to the given sample. The most frequent drugs in the Top 5 for both methods were in cancer-associated drug tiers (Tiers 1–5).

Moreover, for each method, we calculated the proportion of drugs indicated for thyroid cancer (Tiers 1A, 2A, 3A and 4A) within the Top 10 for each THCA-US sample to assess the discriminative capacity of the PANACEA methods. The median proportion of drugs implicated for thyroid cancer was 0.94 (IQR = 0.1) for the ‘distance-based’ method, and 0.8 (IQR = 0.1) for the ‘propagation’ method, indicating high cancer-specific discriminative capacity for both methods.

To investigate the robustness of the methods to the choice of PIN, analyses on THCA-US data were also performed using the BioGRID PIN. The proportions of supported drugs per method were similarly high (Supplementary Figs S5 and S6). Thus, it was concluded that the methods are robust to the choice of PIN, yielding comparable results.

### 3.2 GDSC analysis

The drug prioritization methods were additionally evaluated using drug–response data from GDSC for 18 different cancer types (*n* = 515 cell line samples). The median number of drugs tested per cell line was 382 (interquartile range =55).

**Fig. 4.**
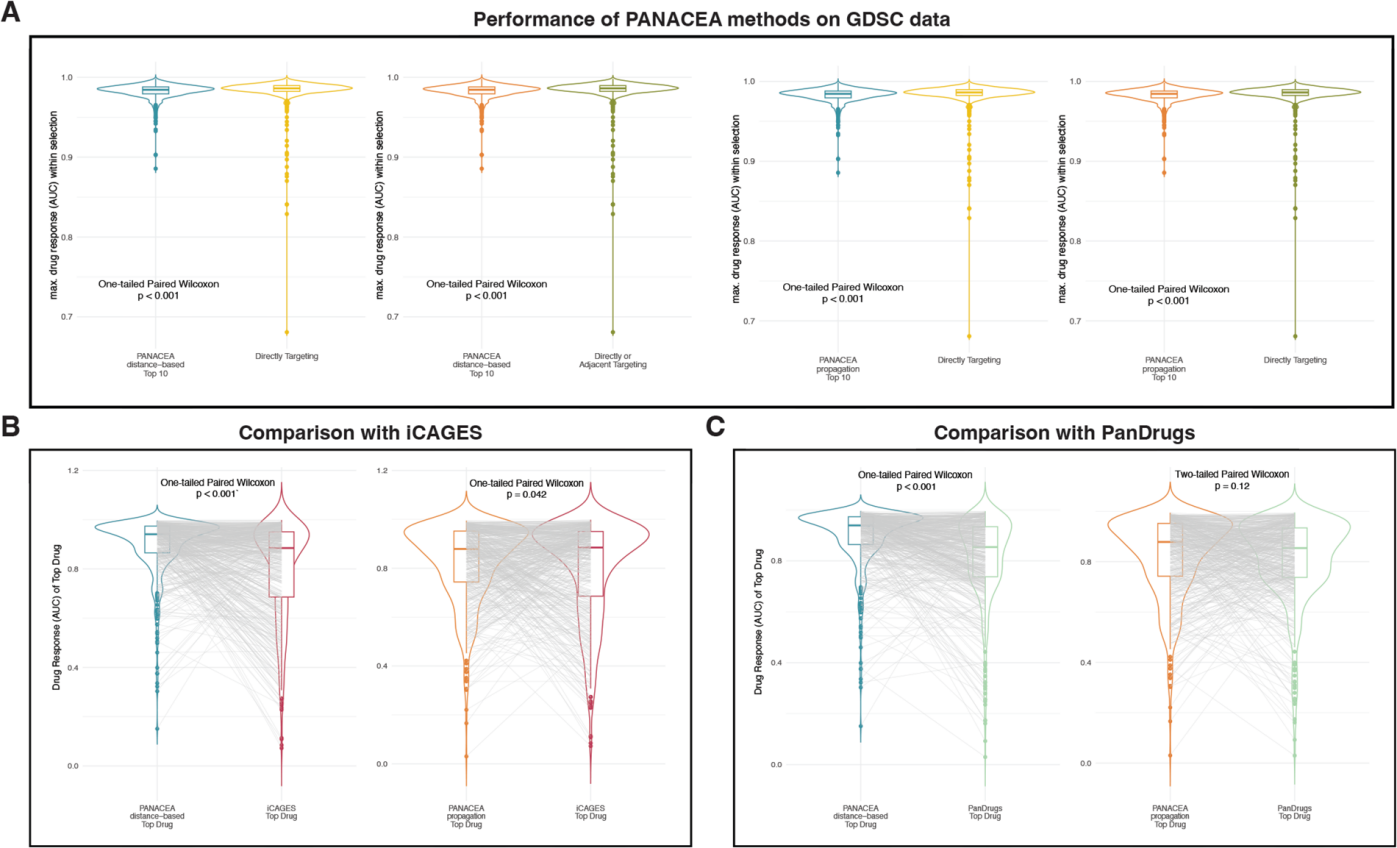
Performance on GDSC cell line drug–response data. (**A**) Comparison of drug response (as measured by AUC) between PANACEA methods and naïve methods (directly targeting and directly or adjacent targeting). (**B**) Comparison of drug response (as measured by AUC) between PANACEA methods—top drug and iCAGES—top drug. (**C**) Comparison of drug response (as measured by AUC) between PANACEA methods—top drug and PanDrugs—top drug

When we compared the PANACEA methods against naïve drug selection approaches (i.e. selecting drugs that directly target driver genes or selecting drugs that directly target driver genes or their direct neighbors), it was observed that the PANACEA methods yielded significantly higher drug response (as measured by the highest drug–response curve AUC within the selected drugs for the screened cell line) (all one-tailed paired Wilcoxon tests *P* < 0.001, Fig. 4A). Moreover, selecting the top drug by each method, the PANACEA methods selected drugs with significantly higher drug response compared to iCAGES (both one-tailed paired Wilcoxon tests *P* < 0.05, Fig. 4B). Similarly, the PANACEA ‘distance-based’ method selected drugs with higher drug response compared to PanDrugs (one-tailed paired Wilcoxon test *P* < 0.001) whereas the drug response of the PANACEA ‘propagation’ method was comparable to PanDrugs (two-tailed paired Wilcoxon test *P* = 0.12, Fig. 4C). When the drug response (AUC) of the top drug by the two PANACEA methods were compared, the ‘distance-based’ method was found to yield a significantly higher AUC compared to the ‘propagation’ method (one-tailed paired Wilcoxon test *P* < 0.001).

The relative frequencies of drugs recommended by each method per cancer type are displayed in Supplementary Figure S7. Especially with the propagation method, it can be observed that the relative frequencies of recommended drugs per cancer type are strongly right-skewed, implying that the majority of recommended drugs are sample-specific.

### 3.3 Cases with known drug response

We applied the PANACEA methods, iCAGES, and PanDrugs on the BLCA and LUAD cases. In the corresponding studies, the BLCA case was reported to be responsive to everolimus and pazopanib, and the LUAD case was reported to be responsive to sorafenib treatment (Imielinski *et al.*, 2014; Wagle *et al.*, 2014).

PANACEA methods successfully recommended drugs known to be effective for both cases (Table 2). While iCAGES successfully recommended sorafenib for the LUAD case and everolimus for the BLCA case, it did not consider pazopanib in the analysis. PanDrugs missed pazopanib for the BLCA case when DScore is used, and sorafenib was not recommended as a ‘Best Candidate Therapy’.

**Table 2.** Recommendation status of effective drugs for the BLCA and LUAD cases per method

	BLCA		LUAD
	Everolimus	Pazopanib	Sorafenib
PANACEA			
Distance-based score—Top 10	Yes	Yes	Yes
Propagation score—Top 10	Yes	Yes	Yes
iCAGES			
iCagesDrugScore—Top 10	Yes	n/a	Yes
PanDrugs			
DScore—Top 10	Yes	No	Yes
Best candidate therapy	Yes	Yes	No

*Note*: ‘n/a’ indicates ‘not available’, i.e. the drug was not considered by the given method.

Full results of all analyses for drug prioritization using the PANACEA methods, iCAGES, and PanDrugs on the BLCA and LUAD cases are provided in Supplementary Tables S4–S11.

### 3.4 The PANACEA R package

The two network-based methods proposed by this study were implemented in an R package available on GitHub (https://github.com/egeulgen/PANACEA.git). Example data (from driveR) are provided and example usage is demonstrated in the vignette ‘How to use PANACEA’ (https://egeulgen.github.io/PANACEA/articles/how_to_use.html). The default drug interactions data are from DGIdb expert-curated sources, and the default PIN is STRING v11.5 interactions with combined scores >0.4 (for moderate confidence, as utilized in this study). Both the drug interaction and PIN data can be customized. The input is a data frame of driveR results, consisting of gene symbols and corresponding driverness scores. The output is the sorted scores of all drugs (a named vector).

The run-times of the two PANACEA methods (distance-based and propagation) as implemented in the R package were analyzed on the TCGA—LAML-US (acute myeloid leukemia, *n* = 135) data (on a computer with Intel Core i9 processor running at 2.9 GHz, 32 GB RAM, MacOS 12.5.1, R version 4.2.1, PANACEA version 0.0.0.9002). The mean run-time of the distance-based method was shorter than the propagation method (Table 3). Additionally, overall, the run-time of the distance-based method was also shorter (as indicated by lower Min., *Q*1, Median, *Q*3 and Max. run-times).

**Table 3.** Summary of run-times for the PANACEA methods on the LAML-US data (*n* = 135 samples)

	Min.	*Q*1	Median	Mean	*Q*3	Max.
Distance-based	42.77	44.41	45.48	45.90	47.33	56.01
Propagation	86.33	91.79	96.55	96.60	100.50	110.33

*Note*: The units are seconds. ‘Min’ and ‘Max’ indicate the minimum and maximum, respectively. ‘Q1’ and ‘Q3’ indicate the first and third quartiles, respectively.

## 4 Discussion

One of the aims of personalized oncology is to leverage information from genomics data, identify clinically relevant alterations and prescribe rational, effective and tailored treatments (Gómez-López *et al.*, 2019). The methods proposed in this study intend to address this aim by incorporating the genomic landscape of tumors, protein interactions and drug–gene interactions from curated sources.

Starting from the list of altered genes in a tumor sample and associated driverness scores, PANACEA methods map these onto the interaction network, along with drug candidates. We would like to note that while driveR is suggested, another method can be utilized to obtain the driverness scores. The ‘distance-based’ method ranks the drugs utilizing the driverness scores and the shortest distance between the altered genes and the drugs. The ‘propagation’ method uses an RWR framework to propagate driverness scores for the same purpose.

Via the network-based methods and a probabilistic approach for defining driverness, we extend the use of genes beyond known drivers and enable each altered gene to be taken into account while computing the drug score. Incorporation of the extended network enables the utilization of indirect interactions between drugs and altered genes. To our knowledge, there is no other method employing a similar approach.

Using multiple datasets, we demonstrated that both the distance-based method and the propagation method of PANACEA can efficiently prioritize clinically appropriate pharmacotherapy options. In the comparisons with two naïve methods and two previous popular approaches, iCAGES and PanDrugs, PANACEA distance-based method outperformed its competitors, and PANACEA propagation method performed as well as or better than them. The major advantage of the PANACEA methods over iCAGES and PanDrugs is that the PANACEA methods extend the utilization of driver genes beyond direct drug targets, as discussed above. Moreover, the PANACEA methods are flexible such that they can be applied using the user’s choice of PIN and drug–target gene resource. The methods are implemented as an easy-to-use R package.

Via analysis of TCGA data using the BioGRID PIN in addition to the STRING PIN, we demonstrate that the PANACEA methods are robust to the change of interaction network.

Notably, the drug options for these methods need not be limited to approved drugs; drugs in clinical trials or even preclinical drugs can also be utilized. This extends the options beyond known anti-cancer drugs, potentially paving the way for drug repurposing, which has gained a great deal of attention in oncology during the last decade (Schein, 2021; Zhang *et al.*, 2020).

Of note, the novel evaluation schemes, we employed in this study can be beneficial in assessing the performance of novel personalized drug prioritization methods by both evaluating the selected drugs’ relevance to cancer treatment (via annotation of approved, clinical trial and preclinical drugs) as well their potential to be efficient therapies (via drug–response data).

Although the PANACEA methods proposed in this study are valuable for enabling personalized drug prioritization, further research is required to improve personalized cancer treatment. Specifically, moving beyond utilizing only genomics data, integrating multi-omics data would allow researchers to have a broader view of the cancer samples’ dependencies influencing response to therapy.

Overall, the PANACEA distance-based method yielded better results compared to the propagation method in terms of relevance and performance in the evaluations. Moreover, the run-time of the distance-based was shorter than the propagation method. Hence, we suggest using the distance-based method.

In summary, we illustrate that the network-based methods for personalized drug prioritization proposed in this study perform well and that the prioritized drugs are relevant to oncotherapy. We hope that the methods can provide further insight into personalized drug prioritization using the genomic landscape of the tumor and help progress personalized cancer research.

## Supplementary Material

btad022_Supplementary_Data

## Data Availability

The data and scripts underlying this article will be shared on reasonable request to the corresponding author.
