# Supplementary material for: PANACEA: network-based methods for pharmacotherapy prioritization in personalized oncology
Source: Bioinformatics. 2023 Jan 12;39(1):btad022. doi: 10.1093/bioinformatics/btad022 (PMC9869653; doi:10.1093/bioinformatics/btad022)
Supplement: btad022_Supplementary_Data [file btad022_supplementary_data.zip › Supplementary_Figures.docx]

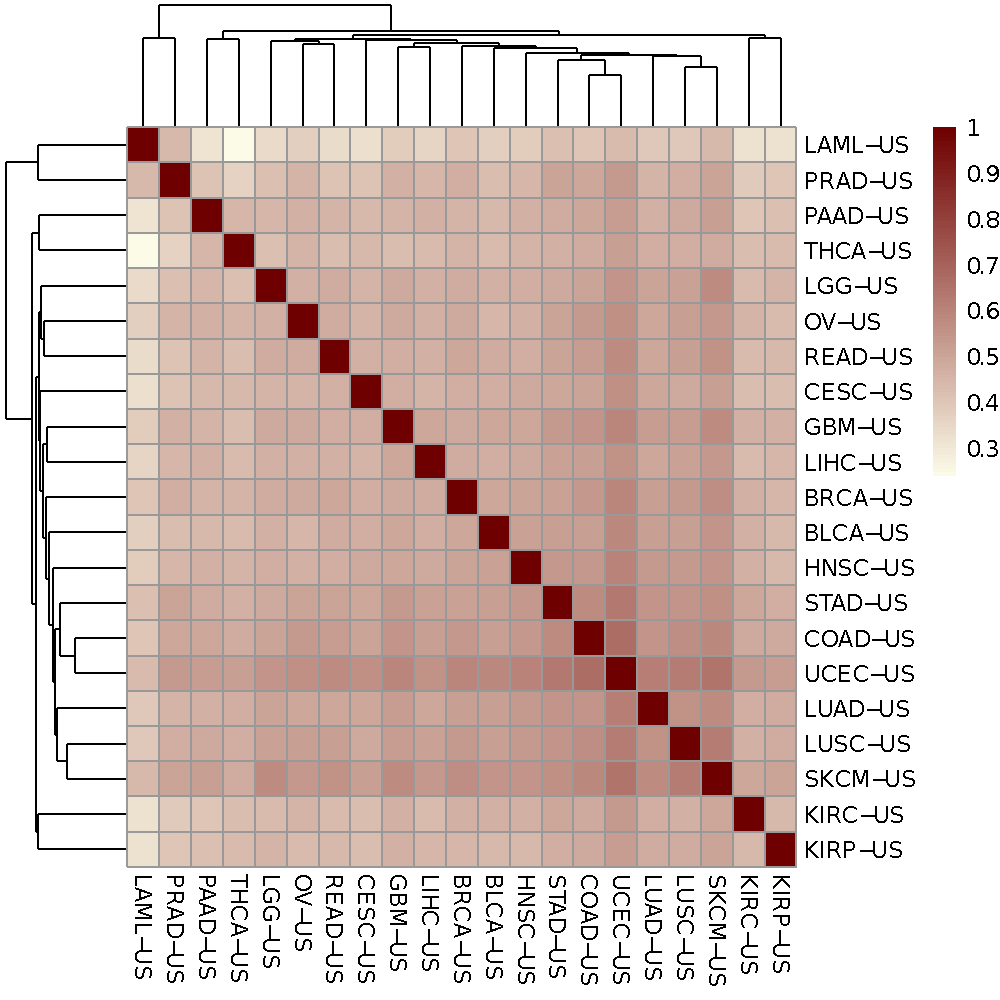


**Figure S1. Clustered heatmap of the Spearman correlation coefficient between each pair of driveR results for selected TCGA cohorts.** The minimum correlation is between LAML-US and THCA-US.

**
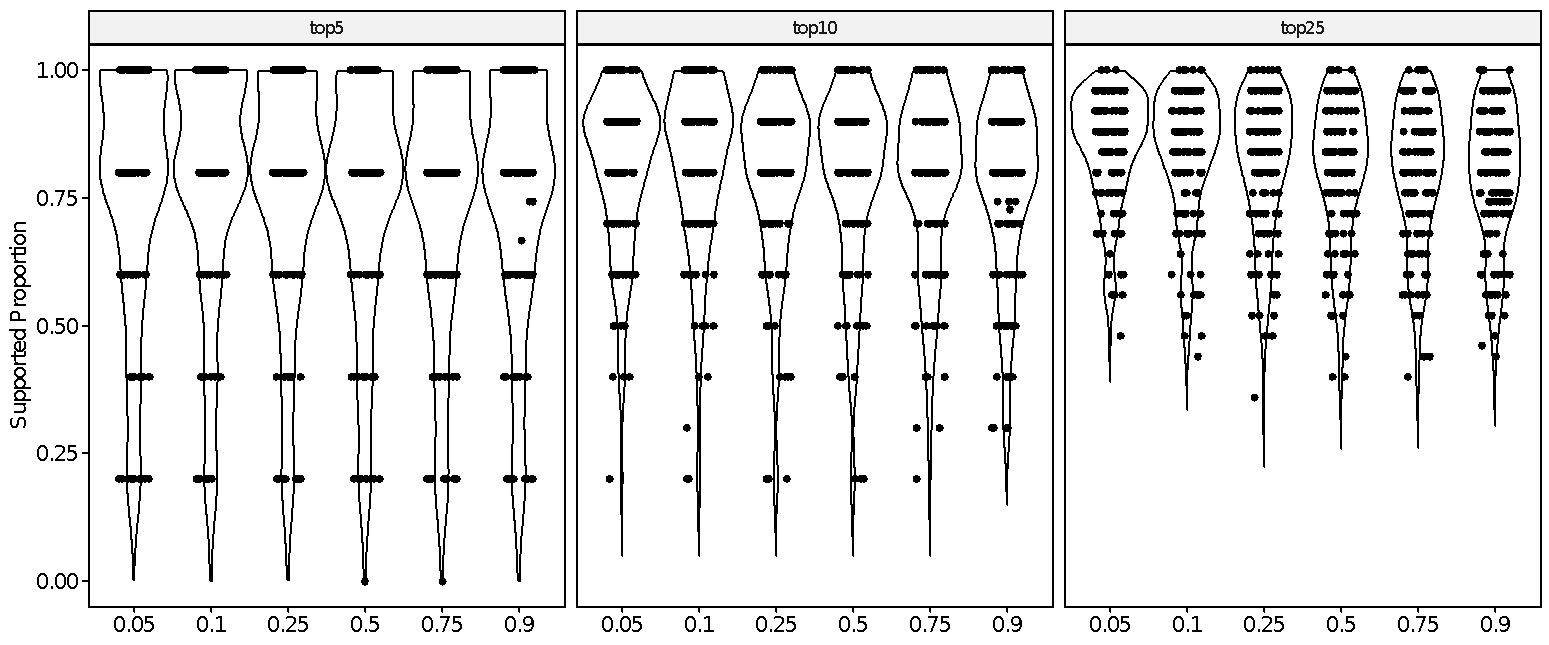
**

**Figure S2. For the propagation method, violin plots displaying the proportion of selected drugs in tiers 1-5 for each choice of the restart parameter (α) per top 5, 10, and 25.** For this parameter selection, the TCGA LAML-US cohort (Acute Myeloid Leukemia) was used. Each dot represents a sample.


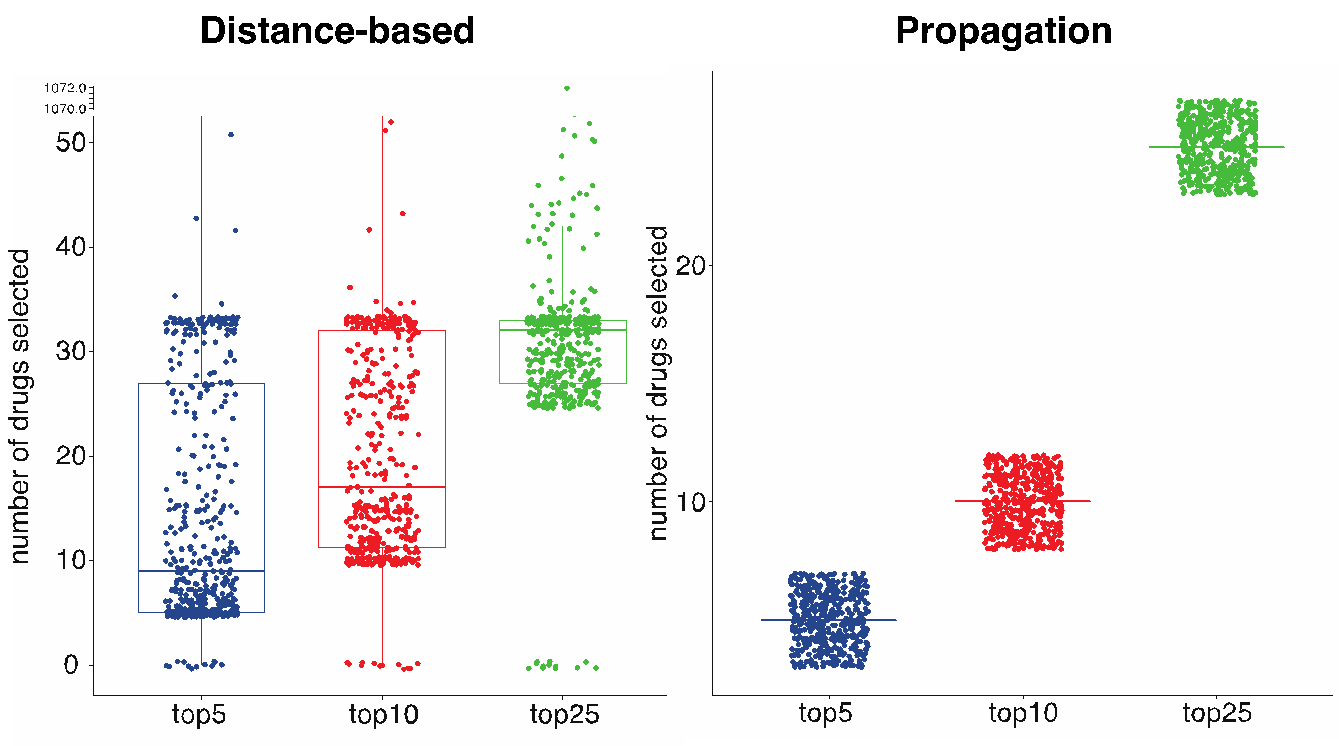


**Figure S3. For each method, boxplots displaying the distribution of the number of drugs selected for the top 5, 10, and 25 (including ties of scores) per sample on TCGA THCA-US data.**

**
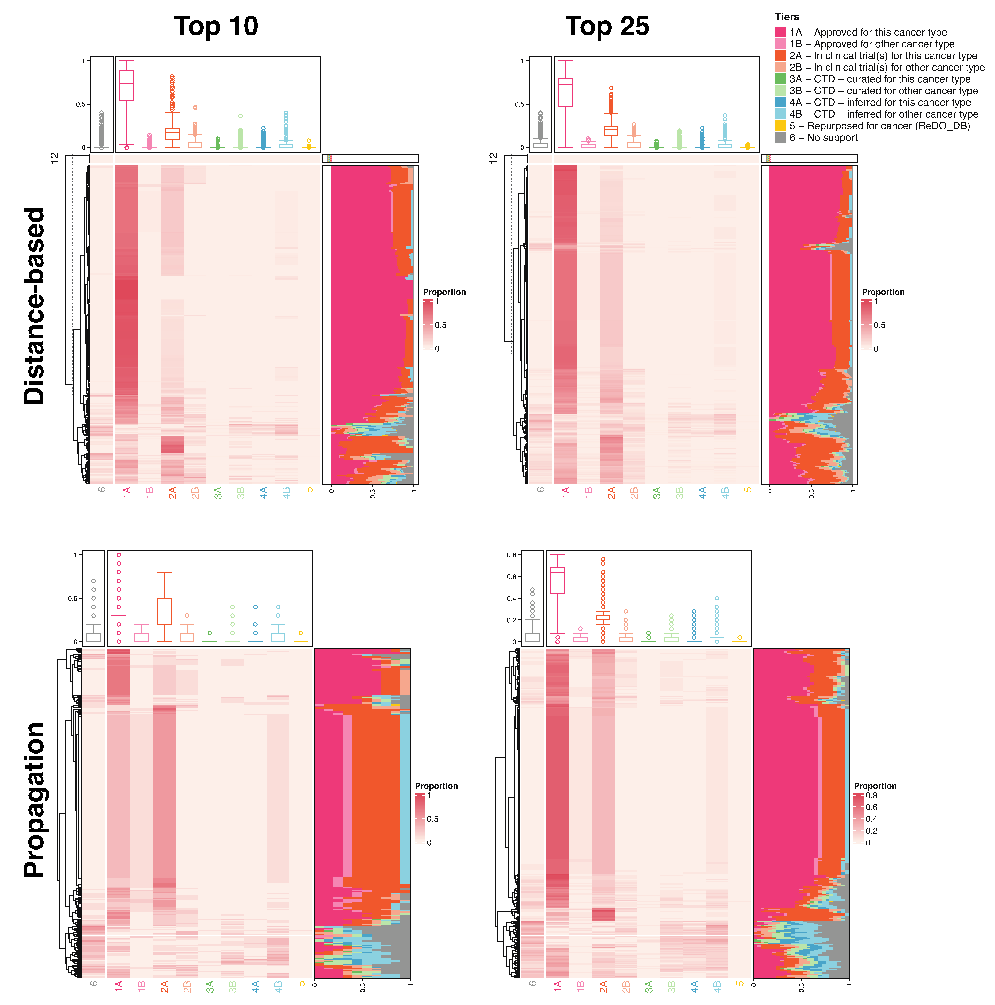
**

**Figure S4. Heatmaps of proportions of selected (top 10 on left and top 25 on right) drugs in each tier for each method (TCGA THCA-US data using the STRING PIN).** For each heatmap, rows are for samples, columns are for drug tiers. Righthand row-side stacked bar plot displays the proportion of selected drugs in each tier per sample. Top box plots display the distributions of proportions of selected drugs per tier. The legend for drug tiers is provided on the top right. For the distance-based method, there were no genes with driveR probability > 0.05 for 12 samples, separately displayed in the heatmaps.

**
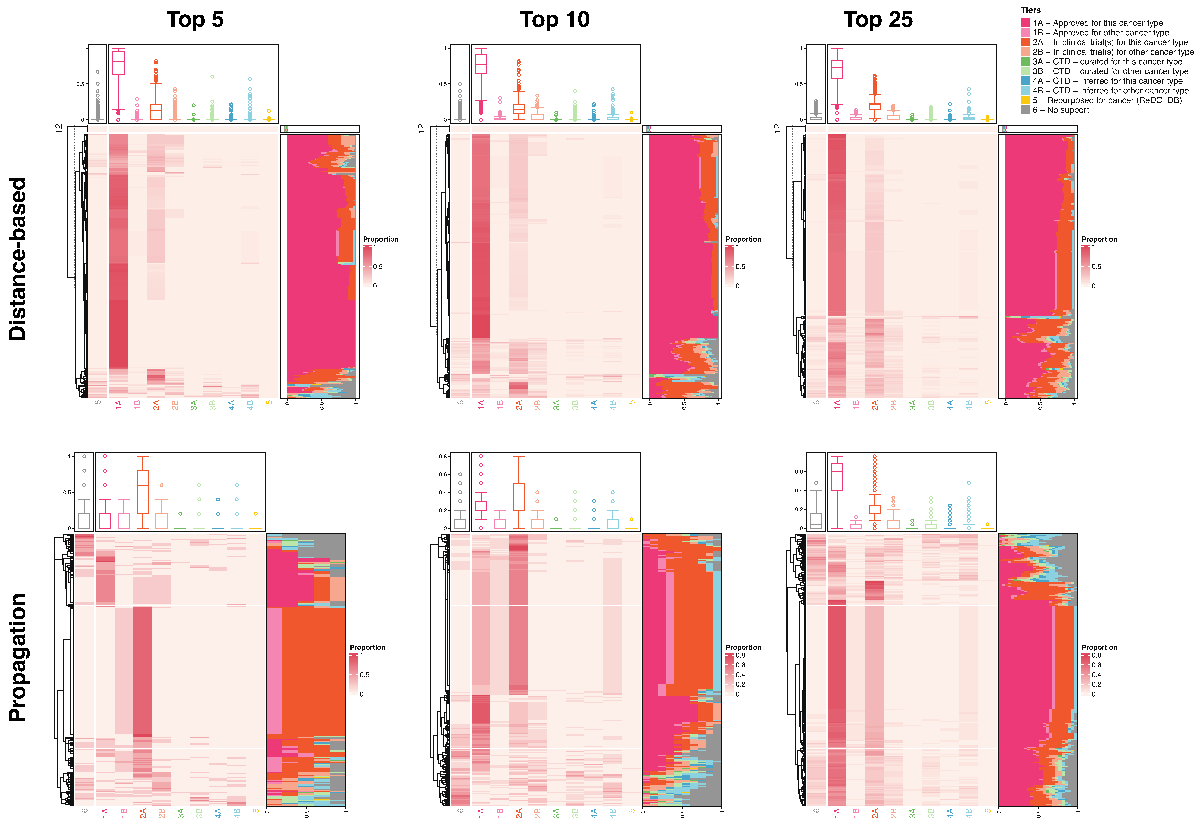
**

**Figure S5. Heatmaps of proportions of selected (top 5 on the left, top 10 in the middle and top 25 on the right) drugs in each tier for each method (TCGA THCA-US data using the BioGRID PIN).** For each heatmap, rows are for samples, columns are for drug tiers. Righthand row-side stacked bar plot displays the proportion of selected drugs in each tier per sample. Top box plots display the distributions of proportions of selected drugs per tier. The legend for drug tiers is provided on the top right. For the distance-based method, there were no genes with driveR probability > 0.05 for 12 samples, separately displayed in the heatmaps.

**
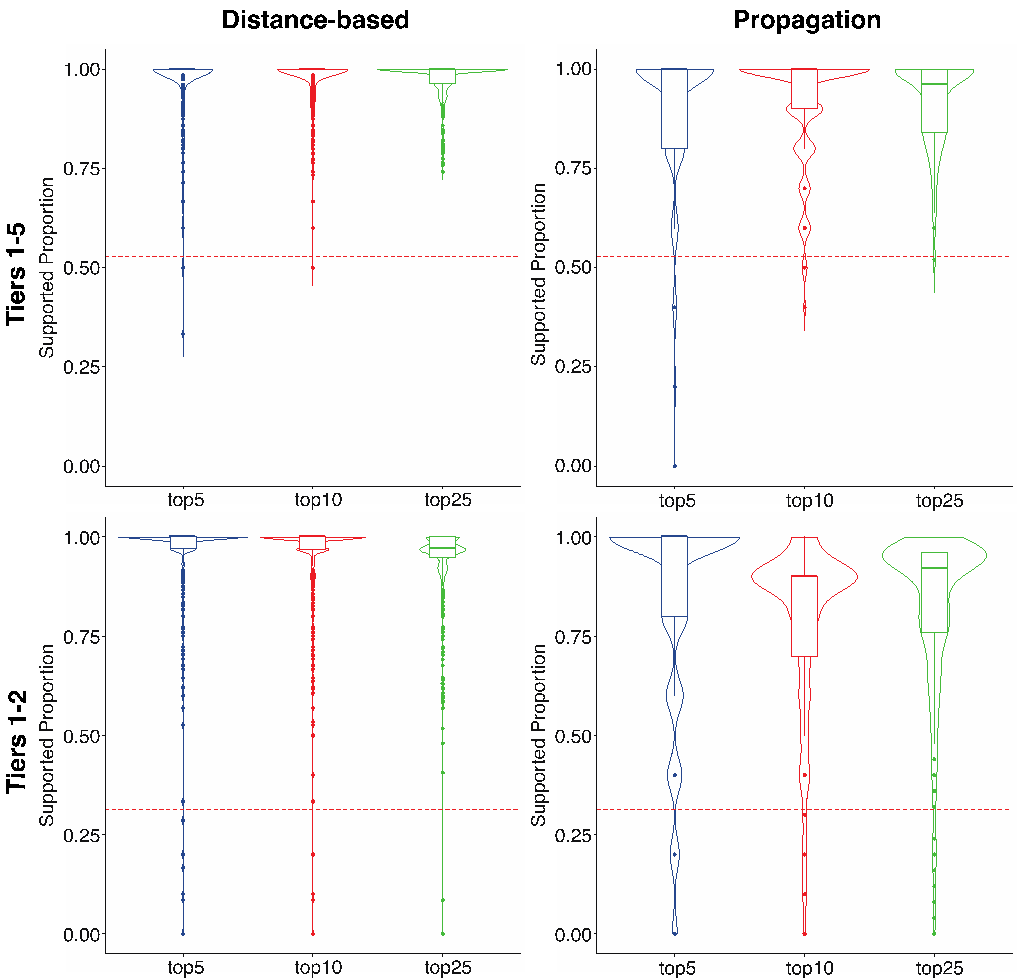
**

**Figure S6. Violin and boxplots displaying the distribution of proportions of supported (either Tiers 1-5 or Tiers 1-2) selected drugs per drug prioritization method (THCA-US data using the BioGRID PIN).** Dashed red lines indicate the overall/expected proportion of tier 1-5 and tier 1-2 drugs in DGIdb.

**
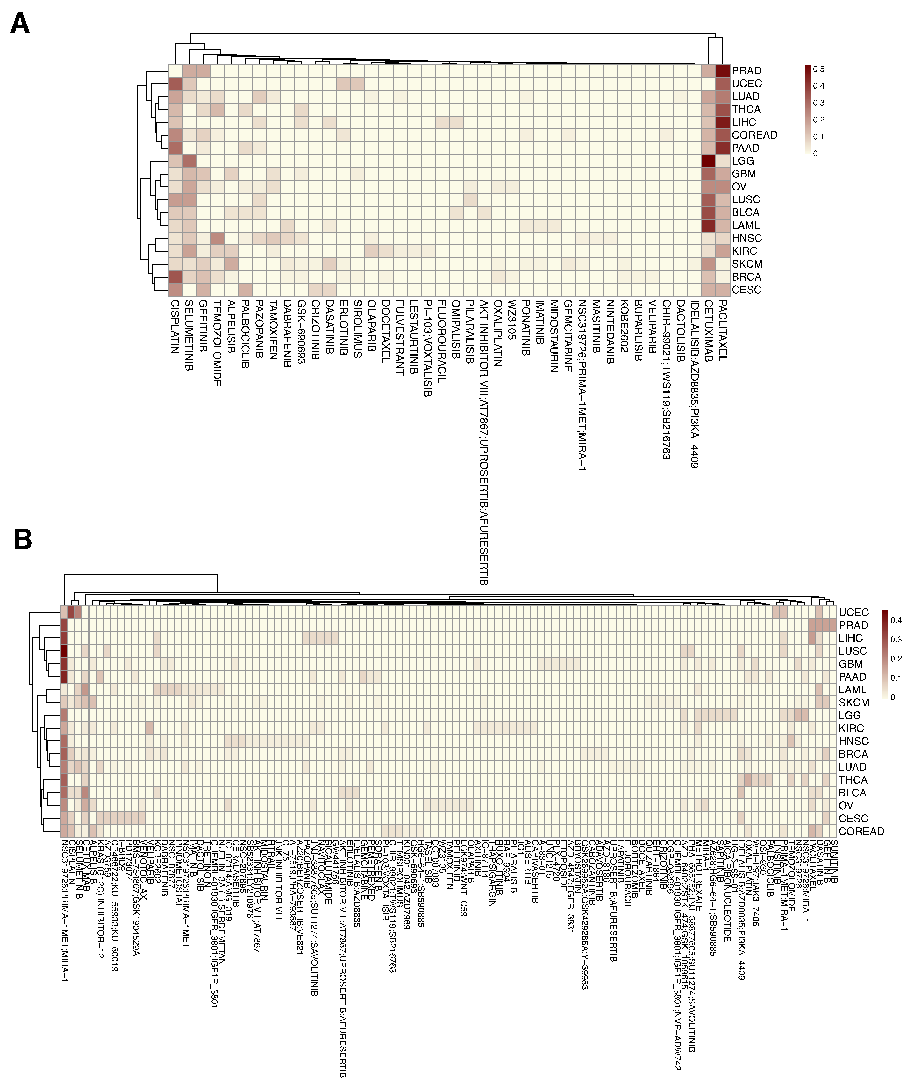
**

**Figure S7. Heatmaps of the relative frequencies of samples in each cancer type (rows) that were recommended a given drug (columns) for the GDSC analysis.** For the recommendation, the drug with the highest AUC value (drug response) among all drugs for the sample was chosen. (A) Distance-based method, (B) Propagation method. Drugs that were merged (because they have the same target gene(s)) are listed separated by ";".
